# Perceptions about reduced antenatal care contacts to a minimum of eight and its associated factors among pregnant women in Bayelsa State, Nigeria

**DOI:** 10.4314/ahs.v25i1.39

**Published:** 2025-03

**Authors:** Olakunle I Makinde, Nkencho Osegi, Adedotun D Adesina, Gordon Atemie, Ninabai N Ofuruma, Chidozie E Unachukwu, Ebiogbo S Ozori, Ariwelo S Warisuo

**Affiliations:** 1 Department of Obstetrics and Gynaecology, Federal Medical Centre, Yenagoa, Bayelsa State, Nigeria; 2 Department of Obstetrics and Gynaecology, Niger-Delta University Teaching Hospital, Okolobiri, Bayelsa State, Nigeria; 3 Department of Medical Services, Nigerian Law School Yenagoa campus, Yenagoa, Bayelsa State

**Keywords:** Antenatal care contacts, Perception, Minimum of eight contacts

## Abstract

**Background:**

Scheduling antenatal care (ANC) contacts in line with WHO-recommended minimum of eight can reduce costs for pregnant women and health systems without compromising quality of care.

**Objectives:**

Assess how pregnant women perceive reduced frequency of scheduled ANC contacts from a minimum of twelve to eight and associated factors.

**Methods:**

Cross-sectional survey of 363 pregnant women receiving ANC in Nigeria. Multivariate logistic regression was conducted using IBM SPSS Statistics version 25; Chicago, IL.

**Results:**

Mean age of respondents was 30.5 ± 5.2 years. Majority had previous ANC experience in at least 1 previous pregnancy (61.7%), were satisfied (79.6%) with the ANC received, and had a negative perception (63.9%; 95%CI: 59.3% - 68.0%) of reduced frequency of scheduled ANC contacts. Satisfaction with ANC received, low social class, and living far from the hospital were the predictors of negative perception.

**Conclusion:**

Women who were satisfied with the traditional model of ANC received, tend to perceive a reduction in the frequency of scheduled ANC contacts negatively. Low social class and living far from the hospital were other predictors of negative perception. Therefore, the characteristics and community features of the population being served should underlie the decision on the frequency of scheduled ANC contacts.

## Introduction

Antenatal care (ANC) is simply defined as a specialized form of care for pregnant women to enable them to attain and maintain a state of good health throughout pregnancy, and to improve their chances of having safe delivery of healthy babies at term^1^.

The traditional ANC model was practised on the assumption that early classification of pregnant women into low-risk and high-risk by predicting complications, and frequent ANC visits were necessary for effective prevention, identification, and treatment of complications in pregnancy^2^. In 1978 the World Health Organization (WHO) integrated a risk-based approach to ANC based on the traditional ANC model^2^. In this model, pregnant women were required to make 12-14 ANC visits regardless of risk status^2^,^3^. However, among other important drawbacks, the traditional ANC model prioritized quantity over quality of care and it failed to accurately identify pregnant women at risk of developing complications. This is because several complications occur late in pregnancy after the risk categorization has been done, consequently, many women previously categorized as low-risk developed complications later in pregnancy^2^,^4^.

In 2002, the WHO recommended the Focused Antenatal Care (FANC) model, a goal-oriented family-centred approach to ANC that prioritizes quality over quantity of care, emphasising women's experience of care. In FANC, targeted maternal and fetal assessment is done at every visit, risk assessment is continued throughout pregnancy, and the decision is made on whether the pregnant woman continues with the basic care component or gets specialized care^5^. Health care providers are to promote the pregnant woman's social and emotional well-being and see her and her spouse as partners in actions and decisions to achieve the desired pregnancy outcome. Rather than the more frequent antenatal visits, a minimum of four quality-focused antenatal clinic contacts with supportive and respectful healthcare providers were prescribed in the basic component of FANC^5^.

Despite improvements in women's experience of care, studies on the impact assessment of FANC showed that four ANC contacts were associated with relatively increased perinatal mortality. Studies also showed that eight ANC contacts reduced perinatal deaths by up to eight per 1000 births compared to four contacts and that there was no important difference between ANC models with a minimum of eight contacts in the basic component and those with more frequent contacts^6^,^7^. In 2016, the FANC model was updated with this new knowledge in a harmonized guideline, prescribing minimum of eight contacts to reduce perinatal mortality and improve women's experience of care. First contact was recommended to have occurred by 12 weeks, and thereafter contacts at 20 and 26 weeks in the 2^nd^ trimester, and 30, 34, 36, 38 and 40 weeks in the third trimester were recommended. Women are to return for delivery at 41 weeks if they have not given birth^8^. With this current recommendation, the discussion about the scheduled frequency of ANC contact has taken two dimensions. That is, moving from a minimum of four ANC contacts to a minimum of eight and bringing the number of contacts down from a minimum of twelve to a minimum of eight.

Nigeria's maternal mortality rate still ranks among the highest in the world and adequate ANC is one of the strategies to reduce these pregnancy-associated deaths or severe maternal morbidities. ANC uptake from a skilled provider in Nigeria increased steadily since 2008, from 58% to 67%, and 57% had at least four ANC contacts by the last demographic and health survey^9^. Cost implication affects provision and utilization of ANC services especially in low- and middle-income countries like Nigeria^10^-^12^. With the new WHO recommendation, ANC services that use the traditional model of ANC contact can reduce the frequency of contacts and cost on pregnant women and health systems without compromising on quality of care and pregnancy outcome. However, ANC attendees' views and expectations should guide policy changes to ensure that maternal satisfaction is not compromised. The tertiary level facilities in Bayelsa State, Nigeria, implement most of the recommendations of the WHO on ANC for a positive pregnancy experience. However, these facilities still adopt the more frequent antennal contacts from the traditional model of ANC (personal communication, June 2021.). The objectives of this study were to assess how pregnant women receiving ANC at the tertiary hospitals in Bayelsa State, Nigeria, perceive a reduction in the frequency of scheduled ANC contacts from a minimum of twelve to eight and associated factors.

## Methods

### Study design

A cross-sectional descriptive questionnaire-based survey.

### Study setting

Bayelsa State is in the South-South geopolitical zone of Nigeria and the state has a population of about 2,700,000. The indigenous people of Bayelsa are collectively referred to as Ijaws and the state is also home to a sizable community of non-indigenous tribes including the Igbos, Ibibios, Efiks, Urhobos, Itsekiris, Isokos, Edos, Yorubas, Hausas etc^13^. Most people in Bayelsa State engage in trading, subsistence farming and small-scale commercial farming. Others work in the State and Federal civil service. Bayelsa State has two tertiary-level health facilities located in Yenagoa; the semi-urban capital city of Bayelsa State, and Okolobiri; a rural small town in Bayelsa State. Apart from being tertiary-level facilities and referral hospitals to peripheral hospitals in Bayelsa State and its environs, these facilities offer ANC primarily. The two centres use the traditional model of ANC contacts. Pregnant women are encouraged to book for ANC by the second missed period, and the follow-up contact schedule for low-risk women in the centres is four-weekly until 28 weeks of gestation, then two-weekly until 36 weeks and thereafter weekly until delivery.

### Inclusion criteria

Pregnant women who attended antenatal clinics at the study centres in July and August 2021 were included.

### Sampling technique

Respondents were selected from the only two tertiary hospitals in Bayelsa State. The total number of respondents per hospital was determined based on the proportion of pregnant women who visit the hospital for ANC per month. Respondents were recruited consecutively in each hospital until the allotted sample size was complete.

### Sample size determination

Using sample size formula for population prevalence^14^,^15^,

N= (Z1-α/2)2 (P) (q) / d2,

where: N= Sample size

Z1-α/2= Level of confidence

P= Prevalence of the dependent variable

q= 1-p, is the acceptable deviation from the true prevalence, and

d= Margin of error; 0.05

By substitution, Z= 1.96 for a confidence interval of 95%

P= 0.653 i.e., 65.3% (a study from the study setting found that 65.3% of the women who had hospital delivery received antenatal care)^16^, and

q= 1-0.653= 0.347

Thus, N= 0.653(0.347) (1.96)2 / 0.052

N= 0.226591(3.8416) / 0.0025

N= 348.19

To control for non-response, 5% was added to the calculated sample size, therefore, N = 366.

## Ethical considerations

Ethical approval was obtained for the study from the institutional research ethics committee. The questionnaire consisted of a consent page which explained the purpose of the research and its benefits and ensured confidentiality by anonymity. Before the questionnaires were administered the women were informed of their right to withhold consent without any form of prejudice from the antenatal service providers. All the respondents gave informed consent to participate in the study.

### Data collection

Data was collected using a structured 3-point scale questionnaire developed by the first author and validated through review by expert obstetricians and statisticians and by pilot test. The questionnaires were mostly self-administered in the English language, and in a few instances, researchers administered the questionnaires in colloquial English. There were five sections on sociodemographic characteristics, previous ANC experience, satisfaction with ANC received, knowledge of the current schedule of ANC contacts being prescribed, and perception of a reduction in the frequency of scheduled ANC contacts to a minimum of eight.

### Data Analysis

Data collected using the questionnaire was checked for correctness and completeness after every data collection exercise. The data was cleaned, coded, and analysed using IBM SPSS Statistics version 25; Chicago, IL, USA.

In the univariate analysis, categorical variables like ethnicity, level of education, marital status, occupation, etc. were summarized as frequencies and percentages, while the continuous variable age was summarized using mean and standard deviation. Parity was summarized using median and range. The factors associated with the perception of a reduction in the frequency of scheduled ANC contacts were assessed using binary logistic regression analysis and variables with significant association (at a p<0.1) were selected into a multivariate logistic regression model. Predictors were identified from multivariate logistic regression at a p<0.05.

The social class of the women was determined using the Oyedeji classification^17^, employing the educational level and occupation of both the respondents (wife) and their spouses. The women and their husbands were scored for their educational attainment and current occupation. Each woman had 4 scores comprising scores for her educational attainment and occupation, and her husband's educational attainment and occupation. The mean of these four scores was calculated, approximated to the nearest whole number, and used to assign a social class to the women from I to V. For this study, social classes I and II were designated as upper social class, III as middle social class, and IV and V as low social class.

The respondents' satisfaction with ANC received (previous and index) in either of the study hospitals was assessed by one item on the questionnaire, with three responses ‘satisfied’, ‘unsatisfied’ and ‘undecided’. The respondents' knowledge of the current schedule of ANC contacts being prescribed was assessed using 6 statements in the questionnaire. Based on an assumption of a normal pregnancy, the respondents were asked to indicate the correctness of statements that presented the schedule of ANC contacts as currently practised in the hospital. The structured responses to the statements were ‘Correct’, ‘Not correct’ and ‘Not sure’. ‘Correct’ was coded 1, while ‘Not Correct’ and ‘Not Sure were coded 0 for all six statements assessing knowledge. With a maximum obtainable point of 6 and a minimum obtainable point of 0, responses were aggregated, and a knowledge score was obtained for each respondent. Respondents with 5-6 points were classified as having good knowledge, 3-4 points as fair knowledge, and <2 points as poor knowledge. The proportion of respondents with good, fair, and poor knowledge was calculated to assess the knowledge level of the current schedule of ANC contacts being prescribed.

Perception of a reduction in the frequency of scheduled ANC contacts to a minimum of eight was assessed using 4 statements in the questionnaire. Based on an assumption of a normal pregnancy, the respondents were asked ‘what do you think?’ about statements that presented the minimal schedule of ANC contacts per trimester of pregnancy as recommended by the WHO. The fourth statement sought their thought on the adequacy of a total of eight contacts throughout a normal pregnancy. The structured responses to these statements were ‘Agree’, ‘Disagree’ and ‘Undecided’. Respondents who agreed to three or all four statements were considered to have a ‘positive perception’. Respondents' perceptions were categorized as ‘equivocal’ when they agreed with only 1 or 2 statements. Those who ‘disagreed’ or were ‘undecided’ with the 4 statements were classified as having ‘negative perception’. To investigate factors related to ‘negative perception’, the variable was re-categorized, and ‘equivocal’ and ‘negative’ perception categories were regrouped as ‘negative perception’.

## Results

### Sociodemographic characteristics of respondents

Three hundred and sixty-six (366) questionnaires were administered and 363 were retrieved, giving a 99.2% response rate. Table 1 shows the sociodemographic characteristics of the respondents.

**Table 1:** Sociodemographic characteristics of respondents

Characteristics	Frequency N = 363	Percent (%)
**Age group**		
< 20 years	7	1.9
20 - 24 years	40	11.0
25 - 29 years	104	28.7
30 - 34 years	125	34.4
35 - 39 years	71	19.6
> 40 years	16	4.4
**Mean Age ± SD in years**	30.5 ± 5.2	
**Ethnicity**		
Ijaw	166	45.7
Igbo	95	26.2
Yoruba	31	8.5
Hausa	6	1.7
Others	65	17.9
**Level of Education**		
No Formal Education	8	2.2
Primary Education	14	3.9
Secondary Education	101	27.8
Tertiary Education	240	66.1
**Marital Status**		
Unmarried	7	1.9
Married	356	98.1
**Residential Location**		
Within Yenagoa/Okolobiri	283	78.0
Outside Yenagoa/Okolobiri	80	22.0
**Occupation**		
Student/Housewife	128	35.3
Pretty Trader/Labourer	80	22.0
Junior Civil servant/Medium scale business owner/Artisan	67	18.5
Professional/Senior civil servant/Large scale business owner	88	24.2
**Social Class**		
Low social class	82	22.6
Middle social class	161	44.4
Upper social class	120	33.1

### Parity, ANC experience and satisfaction with ANC received

Most of the women were multiparous (45.5%), a majority had previous ANC experience from either of the study hospitals in at least 1 previous pregnancy (61.7%) and were satisfied (79.6%) with the ANC received (Table 2).

**Table 2:** Parity, previous ANC experience and satisfaction with ANC received among respondents

Characteristics	Frequency N = 363	Percent (%)
**Parity**		
Nulliparity	103	28.4
Primiparity	75	20.7
Multiparity	165	45.5
Grand-multiparity	20	5.5
**Median parity (Range)**	2 (0 – 6)	
**Previous ANC experience before the index pregnancy**		
Not at all	139	38.3
Yes in 1 previous pregnancy	116	32.0
Yes in > 1 previous pregnancy	108	29.8
**Satisfaction with ANC received**		
Satisfied	289	79.6
Unsatisfied	28	7.7
Undecided	46	12.7

### Knowledge of the current schedule of ANC contacts

Table 3 shows the response pattern to statements assessing knowledge of the current schedule of ANC contacts being prescribed. Most (59.5%) of the respondents had good knowledge of the current schedule of ANC contacts. Another 18.7% had fair knowledge, while 21.8% had poor knowledge.

**Table 3:** Response pattern to statements assessing knowledge of the current schedule of ANC contacts

Question	Response n = 363 (%)			

Statement	Correct	Not Correct	Not Sure	Non-response
You should register for ANC at 2 months of pregnancy even if your pregnancy is going on fine	254 (70.0)	39 (10.7)	64 (17.6)	6 (1.7)
You should attend ANC a minimum of 11-15 times even if your pregnancy is going on fine	245 (67.5)	53 (14.6)	58 (16.0)	7 (1.9)
You should attend ANC at least twice in the 1st 3 months of pregnancy even if your pregnancy is going on fine	256 (70.5)	54 (14.9)	44 (12.1)	9 (2.5)
You should attend ANC every month till 6 months of pregnancy even if your pregnancy is going on fine	248 (68.3)	60 (16.5)	43 (11.8)	12 (3.3)
You should attend ANC every 2 weeks from 6-9 months of pregnancy even if your pregnancy is going on fine	249 (68.6)	43 (11.8)	59 (16.3)	12 (3.3)
You should attend ANC every week from 9 months till delivery even if your pregnancy is going on fine	258 (71.1)	57 (15.7)	38 (10.5)	10 (2.8)

### Perception of a reduction in the frequency of scheduled ANC contacts to a minimum of eight and associated factors

Table 4 shows the response pattern to statements assessing the respondents' perception of a reduction in the frequency of scheduled ANC contacts to a minimum of eight. The majority of the women (63.9%; 95%CI: 59.3% - 68.0%) had a negative perception, while 36.1%; 95%CI: 32.0% - 40.8%) had a positive perception.

**Table 4:** Response pattern to statements assessing the perception of a reduction in the frequency of scheduled ANC contacts to a minimum of eight

Question	Response n = 363 (%)			
	Agree	Disagree	Undecided	Non-response
What do you think about the following?				
One antenatal clinic visit during the 1^st^ 3 months of your pregnancy is ok if your pregnancy is going on fine.	176 (48.5)	124 (34.2)	34 (9.4)	29 (8.0)
Two antenatal clinic visits between 3 months and 6 months of pregnancy are ok if your pregnancy is going on fine.	125 (34.4)	154 (42.4)	54 (14.9)	20 (8.3)
Five antenatal clinic visits from 6 months of pregnancy till your delivery are ok if your pregnancy is going on fine.	140 (38.6)	117 (32.2)	70 (19.3)	36 (9.9)
A total of 8 antenatal clinic visits are enough to monitor you and your baby for safe delivery if your pregnancy is going on fine.	165 (45.5)	104 (28.7)	63 (17.4)	31 (8.5)

The odds of a negative perception of a reduction in the frequency of scheduled ANC contacts to a minimum of eight was significantly increased with low social class, multiparity, previous ANC experience and satisfaction with ANC received (Table 5). Women in the low social class were almost twice as likely to have a negative perception than those in the upper social class (OR=1.99 {1.11 – 3.59}, p=0.021). Multiparous women had a 79% higher likelihood of having a negative perception than nulliparous women (OR=1.79 {1.06 – 3.03}, p=0.029). Women with previous ANC experience had a 78% higher likelihood of having a negative perception than those without ANC experience (OR=1.78 {1.13 – 2.82}, p=0.013).

**Table 5:** Relationship between sociodemographic characteristics of respondents and their perception of a reduction in frequency of scheduled ANC contacts to a minimum of eight

Characteristics	Perception		Crude OR (95% CI)	pValue

	Positive N = 131 (%)	Negative N = 232 (%)		
**Age**				
<20 years	0 (0.0)	7 (100.0)	0.14 (0.01 – 2.55)	0.166
20 – 24 years	13 (32.5)	27 (67.5)	1	
25 – 29 years	41 (39.4)	63 (60.6)	1.35 (0.63 – 2.19)	0.443
30 – 34 years	41 (32.8)	84 (67.2)	1.01 (0.47 – 2.17)	0.972
35 – 39 years	30 (42.3)	41 (57.7)	1.52 (0.68 – 3.42)	0.312
>40 years	6 (37.5)	10 (62.5)	1.25 (0.37 – 4.18)	0.721
**Marital Status**				
Unmarried	0 (0.0)	7 (100.0)	0.11 (0.01 – 2.55)	0.139
Married	131 (36.8)	225 (63.2)	1	
**Tribe**				
Ijaw	61 (36.7)	105 (63.3)	2.91 (0.33 – 25.44)	0.335
Igbo	37 (38.9)	58 (61.1)	3.19 (0.36 – 28.39)	0.298
Yoruba	11 (35.5)	20 (64.5)	2.75 (0.28 – 26.61)	0.382
Hausa	1 (16.7)	5 (83.3)	1	
Others	21 (32.2)	44 (67.7)	2.38 (0.26 – 21.73)	0.440
**Level of Education**				
No Formal	1 (12.5)	7 (87.5)	1	
Primary	7 (50.0)	7 (50.0)	7.00 (0.67 – 72.86)	0.104
Secondary	44 (43.6)	57 (56.4)	5.40 (0.64 – 45.56)	0.121
Tertiary	79 (32.9)	161 (67.1)	3.44 (0.42 – 28.40)	0.252
**Occupation**				
Student/Housewife	47 (36.7)	81 (63.3)	1.12 (0.64 – 1.98)	0.692
Petty trader	35 (43.8)	45 (56.3)	1.50 (0.81 – 2.81)	0.200
Junior Civil servant	19 (28.4)	48 (71.6)	0.77 (0.38 – 1.53)	0.448
Senior civil servant	30 (34.1)	58 (65.9)	1	
**Social Class**				
Low	37 (45.1)	45 (54.9)	1.99 (1.11 – 3.59)	0.021*
Middle	59 (36.6)	102 (63.4)	1.41 (0.85 – 2.33)	0.189
Upper	35 (29.2)	85 (70.8)	1	
**Parity**				
Nulliparity	30 (29.1)	73 (70.9)	1	
Primiparous	21 (28.0)	54 (72.0)	0.95 (0.49 – 1.83)	0.870
Multiparous	70 (42.4)	95 (57.6)	1.79 (1.06 – 3.03)	0.029*
Grand-multiparous	10 (50.0)	10 (50.0)	2.43 (0.92 – 6.45)	0.074
**Previous ANC experience**				
Yes	92 (41.1)	132 (58.9)	1.78 (1.13 – 2.82)	0.013*
No	39 (28.1)	100 (71.9)	1	
**Residential Location**				
Within Hospital Locality	95 (33.6)	188 (66.4)	1	
Distant From Hospital	36 (45.0)	44 (55.0)	1.62 (0.98 – 2.68)	0.051≠
**Satisfaction with ANC received**				
Satisfied	109 (37.7)	180 (62.3)	2.88 (1.29 – 6.39)	0.010*
Unsatisfied	14 (50.0)	14 (50.0)	4.75 (1.64 – 13.75)	0.004*
Undecided	8 (17.4)	38 (82.6)	1	

Petty trader includes Labourer; Junior Civil servant includes medium Scale trader and artisan; Senior civil servant includes Professional; OR – Odd ratio.

* Statistically Significant

≠ p<0.1 included in multivariate logistic regression

Decisions on satisfaction (satisfied or unsatisfied) with ANC received also increased the odds of having a negative perception. An adjusted odds ratio of the various categories of the independent variables showed that satisfaction with ANC received is the strongest predictor of a negative perception of a reduction in the frequency of scheduled ANC contacts to a minimum of eight. Other predictors are low social class and living far from the hospital (Table 6). A test of association between the predictor variables showed that satisfaction with ANC received was significantly associated with respondents' social class (X^2^ = 72.016, pValue < 0.001) and with residential location (X^2^ = 21.971, pValue = 0.001).

**Table 6:** Predictors of a negative perception of a reduction in the frequency of scheduled ANC contacts to a minimum of eight

Characteristics	AOR	95% CI for AOR		pValue

		Min	Max	
**Social Class**				
Low	2.05	1.09	3.85	0.025*
Middle	1.54	0.91	2.61	0.108
Upper	1			
**Parity**				
Nulliparity	1			
Primiparous	0.58	0.24	1.41	0.150
Multiparous	1.01	0.43	2.39	0.872
Grand-multiparous	0.97	0.28	3.40	0.970
**Previous ANC experience**				
Yes	1.66	0.77	3.56	0.195
No	1			
**Residential Location**				
Within Hospital Locality	1			
Distant From Hospital	1.70	1.09	2.90	0.043*
**Satisfaction with ANC received**				
Satisfied	2.46	1.07	5.66	0.034*
Unsatisfied	2.82	0.89	8.99	0.079
Undecided	1			

AOR – Adjusted OR

* Statistically Significant

## Discussion

This study assessed how a population of pregnant women receiving ANC at the tertiary hospitals in Bayelsa state, Nigeria, perceive a reduction in the frequency of scheduled ANC contacts from a minimum of twelve to eight and associated factors. Results showed that the majority of the women had a negative perception of fewer scheduled ANC contacts, and the predictors of negative perception were satisfaction with the ANC received, low social class, and living far from the hospital.

Our finding does agree with previous studies in other parts of Nigeria, in which antenatal clinic attendees preferred the traditional model of ANC contact they had received to one with a reduced frequency of ANC contacts^18^-^21^. More opportunities for expectant mothers to be reassured that all is well, for early detection of a problem where there is one, for learning about pregnancy, for familiarization with healthcare providers, and for social interaction with other pregnant women have been adduced as reasons^18^,^20^,^21^. However, in some other previous studies, some populations of antenatal clinic attendees that had experienced the traditional model of ANC contact, preferred^22^ or would accept^21^ reduced frequency of ANC contacts.

Satisfaction with the traditional model of ANC contact previously received was the strongest predictor of negative perception of fewer scheduled ANC contacts in this study. In addition to previously highlighted reasons for desiring the model, the traditional model of ANC contact was found in a previous study to give women more time away from their routine occupations/home chores^20^. These benefits are perhaps a significant component of women's positive pregnancy experience and a drive for their satisfaction.

Satisfaction with ANC is largely also enhanced by women's individual characteristics and community features^23^. In Lagos Nigeria, Olamijulo et al^22^ found that women with at least a secondary level of education tend to prefer reduced ANC contact. A low level of education was thus fingered as the possible determinant of acceptance of reduced ANC contact. However, in our study and other studies in Nigeria in which most of the study population had a similar minimum level of education^18^,^21^, the preference was for more frequent ANC contacts. As Olamijulo et al^22^ noted, we noticed an urban-rural dichotomy across populations studied in Nigeria. Our study population were semi-urban and rural dwellers, Umeora et al^20^ studied a rural population, while other studies were carried out in Lagos^22^ Enugu^18^ and Port Harcourt^19^,^21^ which are urban areas. The studies in urban areas suggested that being an urban dweller who lives far from the hospital could cause dissatisfaction with frequent ANC contacts. With the hustle and bustle of urban life, convenience associated with reduced ANC contact could easily outweigh the benefits that drive satisfaction with frequent ANC contacts. Convenience, cost, and time constraints were the reasons given by the women who accepted reduced ANC contact in studies conducted in Lagos^22^, Enugu^18^, and Port Harcourt^21^. Thus, rather than their level of education, antenatal attendees' perception of a reduction in the frequency of scheduled ANC contacts, may depend on those other individual characteristics and community features that drive satisfaction with ANC and may favour or disfavour a desire for more frequent ANC contacts.

From the result of this study, low social class was also a predictor of a negative perception of a reduction in the frequency of scheduled ANC contacts. It is likely that the effect of low social class on the respondents' perception, was related to their satisfaction with the previous traditional model of ANC contacts; as from the study results, social class was significantly associated with satisfaction with ANC previously received. However, this contradicts studies like that of Tran et al^24^, which found that adequate use of ANC; in terms of the number of contacts made, was significantly lower among poor women. But then, it is likely that for poor women who would not be able to afford the cost of making all expected ANC contacts, frequent scheduled ANC contacts give more options of contacts they can choose from. Low social class may also play a role in how women value the time away from their routine occupations/home chores, and the opportunity to interact with other expectant mothers that ANC contacts offer. These are subject to further studies.

Residence distant from the hospital was another predictor of a negative perception of a reduction in the frequency of scheduled ANC contacts in this study. In a previous study by Emiru et al^23^, residing far from the hospital was identified as a factor that enhances women's satisfaction with ANC services. They reported higher satisfaction with ANC services among women living far from the hospital. This was attributed to the likelihood that women who live far from the hospital are rural dwellers with limited access to healthcare services, who are more likely to have lower expectations, who tend to place more value on service received and are more likely to report higher levels of satisfaction^23^.

In our study, the hospital settings were semi-urban and rural, and the antenatal attendees who lived outside the hospital locality were usually from even less developed areas with limited access to healthcare services. A significant association was established between the residential location of respondents and satisfaction with the traditional model of ANC previously received. Relying on the finding of Emiru et al^23^ and findings from this study, it can be inferred also that the negative perception of respondents who resided far from the hospital was related to their satisfaction with the previous traditional model of ANC contacts. Beyond the value placed on ANC access by this category of pregnant women^23^ as an underlying factor, it is also likely that being rural dwellers, they are more likely to be of low social class and the factors alluded above to possibly underlie the perception of pregnant women of low social class is also at play in them.

Previous studies in Nigeria that assessed the perception and attitude of pregnant women regarding a reduced frequency of ANC contacts considered four ANC contacts against the traditional model. Perception of, and attitude towards eight ANC contacts may differ from four contacts. Findings from this study largely agree with previous findings in support of the traditional model against a minimum of four ANC contacts. However, for stronger evidence for its adoption or otherwise in our setting, more studies are required on pregnant women's view of the WHO-recommended eight ANC contacts in uncomplicated pregnancies. Available evidence on the attitude of pregnant women of low social class to antenatal clinic attendance is conflicting. The actual effect of financial constraints and rural dwelling on the number of ANC contacts desired by pregnant women needs to be further researched.

## Limitation

The time of data collection coincided with post-COVID periods, this could affect perceptions since women may want to move out more after lockdown periods.

## Conclusion

Women who were satisfied with the traditional model of ANC received, tend to perceive a reduction in the frequency of scheduled ANC contacts negatively in this study. Low social class and living far from the hospital were the other predictors of their negative perception. The implication of this finding for practice and policy-making is that the characteristics and community features of the population being served should underlie the decision to adopt a minimum of eight or continue with the traditional minimum of twelve ANC contacts in uncomplicated pregnancies. There are roles for future research to explore the areas earlier highlighted for stronger evidence. Widespread education of women of reproductive age on the benefits of the WHO recommendation of eight ANC contacts in uncomplicated pregnancies is advocated. This should also form part of health talks in antenatal clinics. This perhaps can engender a lagely positive attitude towards this recommendation and pave the way for channelling healthcare resources towards quality rather than quantity of care.
